# Economic evaluation of early-goal directed therapy for high-risk surgical patients

**DOI:** 10.1186/cc10805

**Published:** 2012-03-20

**Authors:** C Ebm, M Cecconi, H Aya, M Geisen, A Rhodes, M Grounds

**Affiliations:** 1 St George's Healthcare Trust, London, UK

## Introduction

Early goal-directed therapy (EGDT) has been shown to reduce postoperative morbidity and length of hospital stay. Our objective was to analyse the cost-effectiveness of early goal-directed proactive therapy versus standard reactive care in patients at high risk of developing postoperative complications.

## Methos

Patient-level outcome data used were based on a previous randomised, controlled trial. A Markov decision model was constructed to analyse costs and outcomes associated with the use of EGDT. Outcomes assessed were postoperative complications, mortality, quality-adjusted life expectancy (QALY) and incremental costs/QALY.

## Results

The main analysis, based on 28-day survival data of 122 patients, revealed an incremental cost-effectiveness ratio of EGDT of £280.15 per patient. Additional costs of £525.43 per patient associated with EGDT were mainly due to costs related to monitor acquisition and staffing (two additional nurses). These costs were balanced by savings due to the significant reduction in length of stay in the hospital and in the ICU and lower complication rates in the GDT arm (mean expenditures/patient £4,511.25 vs. £5,218.75). This outcome was robust to variations in treatment effect (probability of morbidity and mortality) and sensitive to implementation costs of EGDT. See Table 1.

**Table 1:** 

Outcome	Unit	GDT	Standard
Ward stay	(days)	11 (7 to 15)	14 (11 to 27)
Incr. costs	(£)	525.43	-
Inc. effect	(QALY)	1.88	-
ICER	(£/QALY)	280.15	-

ICER, incremental cost-effectiveness ratio.

## Conclusion

The implementation of EGDT appears clinical and cost-effective. Additional implementation costs will be offset by savings due to a marked decrease in complication rates and hospital length of stay. We conclude that GDT provides significant benefits with respect to both clinical and financial outcomes.

## References

[B1] Pearse R Dawson D Fawcett J Rhodes A Early goal directed therapy after major surgery reduces complications and duration of hospital stay. A randomized, controlled trial Crit Care 2005 9 R687 R693 10.1186/cc3887 16356219 PMC1414018

